# Sleep as a Transdiagnostic Target in Psychiatry: Prebiotics, the Gut–Brain Axis, and the Gap Between Mechanistic Plausibility and Clinical Evidence

**DOI:** 10.3390/nu18142366

**Published:** 2026-07-19

**Authors:** Giuseppe Marano, Elena Lucia Valle, Giulio Carriero, Caterina Brisi, Gianandrea Traversi, Osvaldo Mazza, Gabriele Sani, Marianna Mazza

**Affiliations:** 1 Department of Neuroscience, Head-Neck and Chest, Section of Psychiatry, Fondazione Policlinico Universitario Agostino Gemelli IRCCS, Largo Agostino Gemelli 8, 00168 Rome, Italymariannamazza@hotmail.com (M.M.); 2 Department of Neuroscience, Section of Psychiatry, Università Cattolica del Sacro Cuore, 00168 Rome, Italy; 3 Unit of Medical Genetics, Department of Laboratory Medicine, Ospedale Isola Tiberina-Gemelli Isola, 00186 Rome, Italy; 4 Spine Surgery Department, Bambino Gesù Children’s Hospital IRCCS, 00168 Rome, Italy; osvaldo.mazza1973@hotmail.it

**Keywords:** sleep, prebiotics, gut–brain–sleep axis, circadian rhythms, microbiota, nutrition, mental illness, psychiatric symptoms

## Abstract

Sleep disturbances are highly prevalent across psychiatric disorders and represent both a clinical feature and a potential transdiagnostic therapeutic target. Growing evidence suggests that the gut microbiota may contribute to sleep regulation through immune, metabolic, circadian, and neuroendocrine pathways. Prebiotics, defined as selectively utilized substrates that confer health benefits through modulation of host microorganisms, have received increasing attention as nutritional strategies capable of influencing the gut–brain axis. This narrative review summarizes preclinical and human evidence on prebiotic interventions in relation to sleep-related outcomes and psychiatric symptomatology, with particular attention to short-chain fatty acids, circadian regulation, inflammatory pathways, stress-related hypothalamic–pituitary–adrenal axis activity, and microbial metabolite signaling. Preclinical studies suggest that selected prebiotics may influence sleep architecture, stress resilience, neuroinflammation, and behavioral phenotypes, particularly under conditions of stress or sleep disruption, but translation to human populations remains preliminary. Available clinical studies are limited by small sample sizes, heterogeneous prebiotic formulations, variable doses and intervention durations, inconsistent microbiome methodologies, and frequent reliance on subjective sleep measures rather than polysomnography or actigraphy. Therefore, current evidence supports prebiotics as biologically plausible and generally well-tolerated adjunctive strategies, but not as established treatments for insomnia or psychiatric symptoms. Sleep may provide a clinically meaningful transdiagnostic framework for future nutritional psychiatry research, provided that adequately powered randomized controlled trials integrate objective sleep assessment, standardized microbiome and metabolomic profiling, and clinically relevant psychiatric outcomes.

## 1. Introduction

Sleep is a fundamental biological process involved in emotional regulation, cognitive functioning, and general health. Sleep disturbances are highly prevalent across psychiatric disorders and represent one of the most common clinical features in mental health settings [1,2]. Insomnia, hypersomnia, sleep fragmentation, and circadian dysregulation have been described in mood disorders, anxiety disorders, schizophrenia, and substance use disorders, where they are associated with impaired daytime functioning, reduced quality of life, and poorer clinical outcomes [2,3]. Increasing evidence also indicates that sleep disturbances should not be considered merely secondary symptoms of psychiatric illness, but clinically relevant phenomena that may interact with psychopathology in a reciprocal way and contribute to illness course and relapse vulnerability [1,2]. Accordingly, sleep has emerged as a meaningful therapeutic target in contemporary psychiatric research and practice [1,2,3].

Beyond its high prevalence across diagnostic categories, sleep may be conceptualized as a transdiagnostic dimension in psychiatry. Rather than representing a disorder-specific symptom, sleep disturbance appears to reflect a shared process that cuts across mood, anxiety, psychotic, trauma-related, and substance use disorders [4,5]. This perspective is supported by evidence suggesting that insomnia and related sleep abnormalities are associated with symptom severity across multiple psychopathological domains and may contribute to the maintenance of comorbidity [4]. In addition, transdiagnostic models propose that sleep disturbance is mechanistically relevant because it interacts with emotional regulation, circadian biology, and shared neurobiological systems involved in psychopathology [5]. More recent frameworks further emphasize that sleep dysfunction may represent an embedded transdiagnostic substrate in psychiatric illness, with potential implications for early detection, prognosis, and treatment stratification [6]. These observations support the inclusion of sleep within dimensional and mechanism-based models of mental disorders rather than restricting it to a secondary clinical feature [4,5,6].

**Figure 1:**
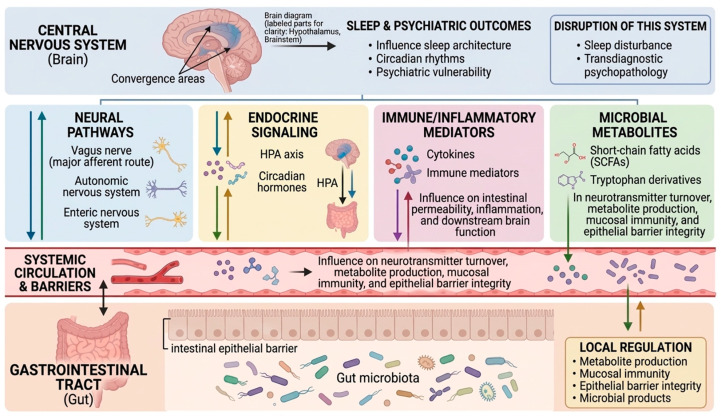
The gut–brain–sleep axis: integrated neural, endocrine, immune, and microbial pathways. Note. The figure illustrates the bidirectional communication between the gut microbiota and the central nervous system in the regulation of sleep. Neural pathways (vagus nerve and autonomic nervous system), endocrine signaling (HPA axis and circadian hormones), immune-inflammatory mediators (cytokines), and microbial metabolites (short-chain fatty acids and tryptophan derivatives) converge to influence sleep architecture, circadian rhythms, and psychiatric vulnerability. Disruption of this system may contribute to sleep disturbance and transdiagnostic psychopathology. Blue Arrows represent neural and classic endocrine signaling pathways (e.g., vagus nerve afferents, HPA axis); Dark Green Arrows signify microbiome-derived communication pathways; Purple Arrows indicate immune-inflammatory mediators and signaling; Brown Arrows denote local, intrinsic regulation within the gut; Red Arrows represent pathways via the systemic circulation.

The gut–brain axis is a complex bidirectional regulatory system linking the gastrointestinal tract and the central nervous system through neural, endocrine, immune, and metabolic pathways [7,8,9]. At the neural level, communication occurs through the enteric nervous system, the autonomic nervous system, and especially the vagus nerve, which represents a major afferent route through which intestinal signals reach the brain [8,9]. Endocrine and neuroendocrine signaling, particularly through the hypothalamic–pituitary–adrenal (HPA) axis, further integrates stress responses with intestinal physiology, while immune mediators and microbial products influence intestinal permeability, inflammation, and downstream brain function [7,8]. In parallel, the gut microbiota contributes to this crosstalk by modulating neurotransmitter turnover, metabolite production, mucosal immunity, and epithelial barrier integrity, thereby shaping both local gastrointestinal homeostasis and systemic neurobiological responses [7,8,9]. Collectively, these mechanisms support the view that the gut–brain axis is not merely an anatomical connection, but a dynamic regulatory network with relevance for both metabolic and neuropsychiatric processes [7,8,9]. The main bidirectional pathways linking the gut–brain axis and sleep regulation are summarized in Figure 1.

The rationale for targeting prebiotics arises from the plasticity of the gut microbiota and the possibility of modulating host physiology through selective nutritional interventions [10,11]. Unlike the human genome, the intestinal microbiome is dynamic and can be reshaped by environmental factors, including diet, making it a plausible therapeutic target [10]. Prebiotics, generally defined as nondigestible substrates such as oligosaccharides that selectively stimulate the growth and activity of beneficial gut microorganisms, may therefore represent a relatively safe and accessible strategy to influence microbiota composition and function [11,12]. Through this mechanism, prebiotics may indirectly affect intestinal barrier integrity, immune regulation, metabolite production, and microbial interactions relevant to gut–brain communication [10,11]. In the context of psychiatric disorders, this approach is particularly attractive because current evidence suggests that microbiota-targeted interventions may help modulate biological pathways implicated in neuropsychiatric symptoms, while offering a low-risk adjunctive option alongside conventional treatments [11,12]. These considerations provide a biological and clinical basis for exploring prebiotics as candidate modulators of the gut–brain axis.

Although several reviews have addressed psychobiotics, probiotics, synbiotics, or microbiota-targeted interventions in psychiatric disorders, the specific contribution of prebiotics to the gut–brain–sleep axis remains less clearly defined. Prebiotics deserve separate consideration because, unlike probiotics, they do not introduce exogenous live microorganisms but act by selectively modifying the activity and metabolic output of resident microbial communities. This distinction is clinically relevant, since different prebiotic compounds may vary substantially in fermentability, microbial targets, short-chain fatty acid production, gastrointestinal tolerability, and potential downstream effects on immune, neuroendocrine, and circadian pathways. Moreover, most previous discussions of microbiota-based interventions in psychiatry have focused primarily on mood, anxiety, or stress-related outcomes, whereas sleep has received less attention as a transdiagnostic therapeutic target. By framing sleep disturbance as a shared clinical and biological dimension across psychiatric disorders, the present review aims to integrate evidence from sleep medicine, nutritional psychiatry, and gut–brain–axis research. This perspective allows prebiotics to be discussed not as stand-alone psychiatric treatments, but as biologically plausible adjunctive strategies whose potential relevance depends on their capacity to influence sleep-related mechanisms that cut across diagnostic boundaries.

In light of these considerations, the aim of this review is to examine whether sleep may represent a clinically relevant transdiagnostic target in psychiatry through modulation of the gut–brain axis, with a particular focus on prebiotics as a potential therapeutic strategy. Specifically, this review summarizes current evidence on sleep disturbances across psychiatric disorders, outlines the main biological mechanisms linking the gut microbiota to sleep and mental health, and discusses preclinical and clinical findings on the effects of prebiotics on sleep-related and psychiatric outcomes. By integrating these lines of evidence, this review seeks to clarify the biological plausibility, current limitations, and potential clinical implications of targeting prebiotics to improve sleep and psychiatric well-being.

## 2. Materials and Methods

This narrative review was conducted to synthesize current evidence on the relationship between sleep disturbances, psychiatric disorders, the gut–brain axis, and prebiotic interventions. The literature was selected with the aim of integrating clinical, preclinical, and mechanistic findings relevant to sleep as a transdiagnostic therapeutic target in psychiatry.

Articles were identified through targeted searches of PubMed, Google Scholar, and reference lists of relevant reviews and original studies. The search focused on combinations of terms related to sleep and psychiatry, including “sleep disturbance”, “insomnia”, “circadian dysregulation”, “psychiatric disorders”, “depression”, “bipolar disorder”, “anxiety”, “PTSD”, “ADHD”, “autism”, “Alzheimer’s disease”, and “Parkinson’s disease”, together with terms related to the gut–brain axis and microbiota-based interventions, including “gut microbiota”, “gut-brain axis”, “prebiotics”, “probiotics”, “synbiotics”, “short-chain fatty acids”, “tryptophan metabolism”, “HPA axis”, “inflammation”, and “neuroinflammation”.

Priority was given to peer-reviewed articles published in English, including systematic reviews, meta-analyses, randomized controlled trials, observational studies, and relevant preclinical studies. Studies were selected according to their relevance to the conceptual framework of the review, with particular attention to evidence linking microbiota modulation, sleep outcomes, stress regulation, inflammation, and psychiatric symptoms. No formal meta-analysis was performed because of the heterogeneity of populations, interventions, outcome measures, and study designs. The findings were therefore organized narratively into thematic sections covering psychiatric sleep disturbances, gut–brain–sleep mechanisms, prebiotic definitions and biological effects, preclinical and human evidence, clinical implications, and future directions.

Illustrative figures were generated using the generative artificial intelligence tool Google Gemini (Gemini 3 Flash, May 2026 version) under the authors’ technical guidance, prompt engineering, and conceptual direction. All AI-generated visual elements were thoroughly reviewed, edited, and verified by the authors to ensure full scientific accuracy and alignment with the study’s framework.

## 3. Sleep Disturbances Across Psychiatric Disorders

### 3.1. Mood Disorders

Sleep disturbances are highly prevalent across mood disorders and encompass insomnia, hypersomnia, poor sleep quality, altered sleep architecture, and circadian dysregulation [13,14,15,16]. This is consistent with broader evidence indicating that sleep abnormalities are among the most common and clinically relevant features across psychiatric disorders, while also showing particular diagnostic and prognostic importance in affective illness [1,2]. In major depressive disorder, sleep complaints are reported by up to 90% of patients, with insomnia representing the most frequent presentation and hypersomnia occurring less commonly but remaining clinically relevant in specific subgroups [13]. Clinically, depressive states are associated with difficulties in sleep initiation and maintenance, early morning awakenings, non-restorative sleep, daytime fatigue, and excessive sleepiness [13,14]. Objective studies further indicate impaired sleep continuity, reduced slow-wave sleep, shortened rapid eye movement (REM) latency, and increased REM density, suggesting that sleep disturbance in depression extends beyond subjective complaints and involves measurable alterations in sleep architecture [13,15]. Importantly, longitudinal evidence indicates that insomnia is not merely a concomitant feature of depression but an independent risk factor for its onset, recurrence, and poorer clinical outcome [15]. Residual sleep disturbance is also common after symptomatic remission and has been associated with increased risk of relapse, highlighting the clinical relevance of directly addressing sleep in patients with depressive disorders [13,15].

In bipolar disorder, sleep–wake disturbance is equally central but often presents with greater variability across illness phases. Manic and hypomanic states are characteristically associated with a reduced need for sleep, whereas depressive episodes may involve either insomnia or hypersomnia [13]. Importantly, sleep disturbance frequently persists during interepisode phases, suggesting that it is not only state-dependent but also a trait-like feature of bipolar illness [13,16]. A systematic review and meta-analysis found that individuals with interepisode bipolar disorder showed longer sleep onset latency, greater wake after sleep onset, more severe insomnia symptoms, greater daytime sleepiness, and increased variability in sleep–wake schedules compared with healthy controls, with some aspects approaching the severity observed in primary insomnia [16]. These findings indicate that bipolar disorder is characterized not only by insomnia and hypersomnia, but also by circadian and rest-activity dysregulation, including unstable daily rhythms and irregular sleep timing, which may contribute to symptom persistence and relapse vulnerability [13,16]. Clinically, this is particularly relevant because sleep disturbance in euthymic bipolar patients has been associated with poorer functioning, greater symptom burden, and increased risk of relapse [13,16]. Overall, the evidence supports the view that sleep abnormalities in mood disorders are pervasive, clinically meaningful, and closely linked to illness trajectory rather than being limited to secondary symptom expression [1,2,13,14,15,16].

### 3.2. Anxiety and Stress-Related Disorders

Sleep disturbances are highly prevalent in anxiety and stress-related disorders and are increasingly recognized as clinically meaningful components of symptom expression, maintenance, and severity [1,5,17,18,19,20,21]. Across these conditions, insomnia appears to be particularly prominent, although disorder-specific patterns such as nightmares, hyperarousal-related sleep fragmentation, and non-restorative sleep are also frequently observed [5,17,18,19,20,21].

In generalized anxiety disorder (GAD), sleep disturbance is among the most frequent and impairing clinical features, with insomnia occupying a central role [17,18,19]. A recent systematic review and meta-analysis reported a pooled prevalence of sleep disturbance of 72% in patients with GAD, confirming that sleep problems are highly common in this population [17]. Clinically, individuals with GAD frequently report difficulty initiating sleep, maintaining sleep, and achieving restorative rest, and insomnia severity appears to be closely associated with anxiety severity, depressive symptoms, pain, and reduced quality of life [17]. This is consistent with broader clinical and neurobiological models suggesting a bidirectional relationship between anxiety and insomnia, whereby cognitive hyperarousal, excessive worry, and physiological tension disrupt sleep, while poor sleep in turn amplifies emotional dysregulation and anxiety symptoms [18]. Earlier clinical literature similarly described sleep-maintenance insomnia as the most characteristic form of sleep disruption in mild-to-moderate GAD, with sleep-onset insomnia also occurring to a lesser extent [19]. Taken together, these findings support the view that insomnia in GAD is not simply an accessory complaint, but a core and potentially perpetuating feature of the disorder [17,18,19].

Sleep disruption is also highly prevalent in PTSD and often includes both insomnia and trauma-related nightmares [20,21]. Subjective studies indicate that 70–91% of patients with PTSD report difficulty falling or staying asleep, while nightmares are also frequently described and may vary according to symptom severity and trauma characteristics [20]. Beyond their high prevalence, sleep problems in PTSD appear closely linked to the disorder’s hyperarousal dimension. In a polysomnographic study, PTSD patients with more prominent hyperarousal symptoms showed poorer sleep efficiency, greater wake after sleep onset, and worse subjective sleep quality than PTSD patients without marked hyperarousal, suggesting that hyperarousal may help explain variability in sleep findings across PTSD samples [21]. This observation is clinically important because insomnia, nightmares, and other forms of fragmented sleep may contribute not only to distress and daytime dysfunction, but also to the maintenance and worsening of PTSD symptoms over time [20,21]. Overall, the evidence suggests that sleep disturbances in PTSD are not merely secondary manifestations of trauma-related distress, but central features that interact dynamically with hyperarousal and symptom persistence [20,21].

### 3.3. Neurodevelopmental and Neurodegenerative Disorders

Sleep disturbances are also highly prevalent in neurodevelopmental and neurodegenerative disorders, where they may contribute not only to symptom burden and reduced quality of life, but also to impaired cognition, behavior, caregiver strain, and disease progression [22,23,24,25,26,27,28,29]. Although the specific sleep phenotype varies across conditions, common patterns include insomnia, circadian disruption, excessive daytime sleepiness, parasomnias, and fragmented sleep [22,23,24,25,26,27,28,29].

Individuals with attention-deficit/hyperactivity disorder (ADHD) frequently experience sleep disturbances, daytime sleepiness, and circadian rhythm abnormalities [22,23]. Across the lifespan, ADHD has been associated with shorter sleep duration, greater daytime sleepiness, increased sleep disturbance, and insufficient sleep relative to same-age peers [22]. Clinical reviews also indicate that the sleep phenotype in ADHD is heterogeneous and may include sleep-onset delay, increased nocturnal motor activity, sleep-disordered breathing, restless legs syndrome, periodic limb movements in sleep, and deficits in alertness [23]. Importantly, these sleep problems cannot be explained solely by medication effects, as children and adolescents with ADHD show higher rates of both subjective and objective sleep impairment even when medication use is taken into account [22,23]. Overall, the evidence suggests that sleep problems are a frequent and clinically relevant component of ADHD rather than a secondary or incidental finding [22,23].

Sleep problems are also extremely common in autism spectrum disorder (ASD), with prevalence estimates in pediatric populations ranging from 40% to 80% [24]. The most frequently reported sleep difficulties include insomnia, parasomnias, and circadian rhythm sleep–wake disorders, often in the context of co-occurring sensory sensitivities, behavioral rigidity, anxiety, and medical contributors such as gastrointestinal symptoms or epilepsy [24]. Poor sleep in autistic children has been linked to increased internalizing and externalizing behaviors, reduced adaptive functioning, and a lower quality of life for both patients and caregivers [24]. More broadly, sleep difficulties are recognized as a frequent co-occurring condition in ASD and should be considered part of its wider clinical burden, alongside other psychiatric and neurologic comorbidities [25]. These observations support routine screening and management of sleep problems in autistic youth as part of standard clinical care [24,25].

Sleep disturbances are highly prevalent in both Alzheimer’s disease and Parkinson’s disease, where REM sleep abnormalities and circadian disruption are especially relevant [26,27,28,29]. In Alzheimer’s disease, patients commonly exhibit fragmented nocturnal sleep, reduced sleep efficiency, increased daytime napping, and, in more advanced stages, marked disruption of the sleep–wake cycle, including circadian misalignment and even day-night reversal [26,27]. Beyond these macrostructural changes, alterations in REM sleep and other sleep stages have been linked to cognitive decline, while accumulating evidence suggests that disturbed sleep may also contribute to Alzheimer’s disease pathogenesis through mechanisms involving inflammation and increased β-amyloid burden [26,27]. This supports a bidirectional model in which neurodegeneration worsens sleep organization, while chronic sleep and circadian disruption may in turn accelerate cognitive deterioration [26,27].

In Parkinson’s disease, sleep disorders encompass insomnia, excessive daytime sleepiness, circadian dysfunction, and parasomnias, with rapid eye movement sleep behavior disorder (RBD) representing one of the most characteristic REM sleep disturbances [28,29]. RBD is of particular importance because it may precede the onset of parkinsonism by several years and is increasingly recognized as a prodromal marker of synucleinopathy [29]. In addition, circadian and sleep–wake regulation are frequently disrupted in Parkinson’s disease as a result of the neurodegenerative process itself, as well as nocturnal motor symptoms, medications, and comorbid conditions that impair sleep continuity and quality [28,29]. Taken together, these findings indicate that in both Alzheimer’s disease and Parkinson’s disease, REM sleep abnormalities and circadian dysregulation are not only common clinical features but also potentially informative markers of disease mechanisms and progression [26,27,28,29].

## 4. The Gut–Brain–Sleep Axis

### 4.1. Microbiota Composition and Circadian Rhythms

The gut microbiota exhibits marked circadian variation in both composition and function, with a substantial proportion of microbial taxa and metabolites fluctuating rhythmically across the day [30,31,32,33]. This temporal organization fits within the broader framework of the gut–brain axis, a bidirectional regulatory system linking intestinal, neural, endocrine, immune, and metabolic pathways [8,9]. Current evidence indicates that microbial oscillations are closely linked to host circadian organization and are shaped by light-dark cycles, feeding-fasting rhythms, meal timing, and diet composition [30,31,32]. Under physiological conditions, this temporal structure appears to support metabolic flexibility and host homeostasis by aligning microbial activity with predictable changes in nutrient availability and host behavior [30,31].

It is important to stress that the relationship between circadian rhythms and the gut microbiota is bidirectional. On the one hand, host circadian clocks influence microbial structure, abundance, and function; on the other hand, the microbiota contributes to the synchronization of peripheral circadian pathways through microbial metabolites and signaling molecules, including short-chain fatty acids and other products of bacterial fermentation [30,31,32,33]. Experimental studies have shown that disruption of circadian rhythms induced by altered light exposure, clock gene dysfunction, shift-like conditions, or irregular feeding schedules can dampen microbial rhythmicity, alter taxonomic composition, and impair microbial functions relevant to intestinal barrier integrity, immune regulation, and metabolism [30,31,32,33]. These findings support the idea that circadian misalignment is associated not only with changes in the abundance of specific taxa but also with a broader loss of temporal organization in microbiota function [30,31,32].

Diet appears to play a pivotal role in this interaction. Meal timing and dietary composition can act as major entraining signals for peripheral clocks and simultaneously influence the oscillatory behavior of the gut microbiota [30,31,32]. In this sense, the microbiota may be viewed as a temporally responsive component of the gut–brain axis, integrating circadian, nutritional, and metabolic information [8,9,33]. When this finely regulated system becomes desynchronized, the resulting dysbiosis and loss of rhythmic microbial signaling may contribute to inflammation, metabolic dysfunction, and possibly neurobehavioral disturbances [30,33]. Overall, these observations suggest that microbiota composition is not static but dynamically organized across the day, and that preservation of microbial rhythmicity may be relevant for both systemic and mental health [30,31,32,33].

### 4.2. Neuroendocrine Pathways

**Figure 2:**
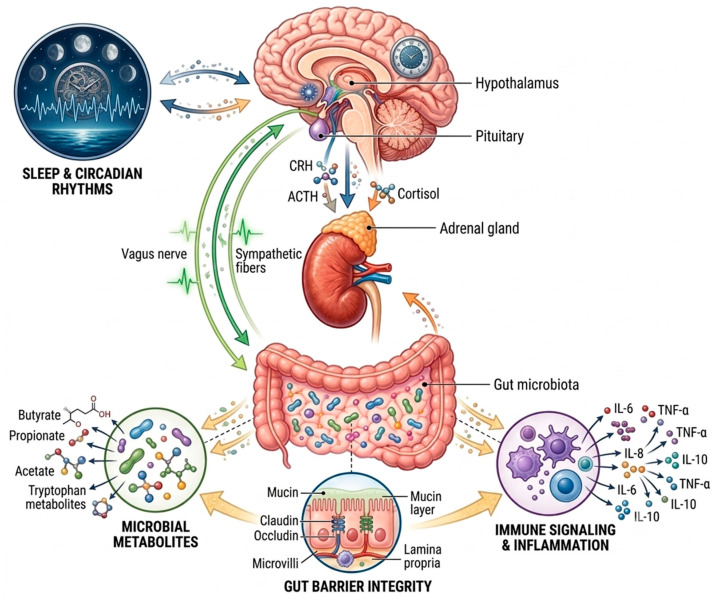
Neuroendocrine pathways linking the gut–brain axis, stress regulation, and sleep. Note. The figure summarizes the interaction between hypothalamic–pituitary–adrenal axis activity, cortisol rhythmicity, melatonin secretion, gut microbiota signaling, immune activation, and sleep–wake regulation. It emphasizes how stress-related and circadian hormonal signals may converge on sleep continuity, arousal regulation, and psychiatric vulnerability. Green arrows represent bidirectional autonomic neural pathways, differentiating the vagus nerve (parasympathetic afferents/efferents) and sympathetic fibers. Blue and Orange central arrows illustrate the neuroendocrine cascade of the HPA axis (CRH/ACTH signaling) leading to systemic cortisol release. Purple arrows indicate immune signaling cascades and the systemic release of pro- and anti-inflammatory cytokines (IL-6, TNF-alpha, IL-8, IL-10). Yellow/Orange gradient arrows represent the bidirectional trafficking of microbial metabolites, gut barrier integrity crosstalk, and local biochemical signaling. Blue/Orange top-left arrows denote the reciprocal feedback loops between central sleep-circadian centers and peripheral physiological rhythms.

Neuroendocrine pathways represent a key interface through which the gut–brain axis may influence sleep regulation and psychiatric functioning. Among these pathways, the HPA axis and the circadian organization of melatonin and cortisol secretion are particularly relevant, as they link stress physiology, sleep architecture, and circadian timing [34,35,36,37,38,39]. Figure 2 provides a schematic overview of this neuroendocrine interface, highlighting the temporal coordination between stress-related and circadian hormonal signals.

The HPA axis is a major neuroendocrine system involved in stress adaptation and follows a robust circadian rhythm, with low nocturnal cortisol levels, a rise during the latter part of the sleep period, and a peak in the early morning [34,35]. Under physiological conditions, sleep onset is associated with a relative inhibition of HPA activity, whereas awakenings, sleep fragmentation, and sleep offset are accompanied by stimulation of cortisol secretion [35]. This close temporal coupling indicates that sleep and HPA function are reciprocally regulated rather than operating as independent systems [34,35].

Evidence further suggests that inadequate or disturbed sleep may alter not only basal HPA activity but also its responsiveness to stress. A systematic review of human studies concluded that both objective and subjective reductions in sleep quality tend to potentiate cortisol reactivity to laboratory stress, supporting the view that poor sleep sensitizes the HPA axis [36]. In parallel, chronic sleep disruption and insomnia have been associated with HPA hyperactivity, increased arousal, and impaired recovery from stress, mechanisms that may contribute to both psychiatric vulnerability and sleep maintenance difficulties [34,35]. Within the broader gut–brain framework, this is particularly relevant because stress-related microbial and immune signals may converge on HPA regulation, thereby reinforcing a vicious cycle between dysbiosis, neuroendocrine activation, and disturbed sleep.

Alongside the HPA axis, melatonin and cortisol represent two major hormonal signals involved in circadian sleep regulation. Melatonin, released primarily during the biological night, contributes to circadian synchronization and facilitates sleep onset, duration, and sleep quality, whereas cortisol displays an opposing rhythm that promotes wakefulness and peaks in the morning [37,39]. This temporal opposition is central to the normal organization of the sleep–wake cycle and may become disrupted in individuals with insomnia, circadian misalignment, or chronic stress [37,39].

Alterations in cortisol secretion also appear to reflect the subjective and physiological burden of poor sleep. In patients with primary insomnia, awakening salivary cortisol has been found to correlate with sleep disturbance parameters, including poorer sleep quality, more frequent nocturnal awakenings, and reduced restoration after sleep [38]. In addition, chronotype-related differences in biological timing have been described for both cortisol and melatonin, further supporting the idea that individual circadian organization influences sleep propensity, alertness, and vulnerability to misalignment [39]. The neuroendocrine regulation of sleep depends on a finely coordinated balance between stress-related HPA activation and circadian hormonal signals, and that disruption of this balance may be relevant to both sleep disturbance and psychiatric symptomatology [34,35,36,37,38,39].

### 4.3. Immune and Inflammatory Mechanisms

Immune and inflammatory signaling represents an important pathway linking sleep, circadian regulation, and the gut–brain axis. Under physiological conditions, inflammatory activity is not constant across the 24 h cycle, but varies according to both circadian timing and sleep state. Conversely, sleep disturbance can promote a pro-inflammatory profile that may alter sleep continuity, sleep architecture, and daytime functioning, supporting a bidirectional relationship between sleep and immune activation [40,41,42,43,44].

Among the inflammatory mediators most consistently implicated in sleep regulation are interleukin-6 (IL-6), tumor necrosis factor-alpha (TNF-α), and interleukin-1β (IL-1β) [41,42,43]. These cytokines show circadian variation and are modulated by both normal sleep and sleep loss. Experimental and clinical evidence indicates that sleep deprivation or fragmented sleep can shift cytokine secretion toward a more pronounced daytime inflammatory profile, whereas normal nocturnal sleep contributes to the maintenance of inflammatory timing [42,43]. In addition, TNF-α and IL-1β appear to act not only as markers of sleep disruption but also as active regulators of sleep physiology, especially in relation to non-rapid eye movement sleep and sleep homeostasis [41,42]. Even in individuals without a diagnosed sleep disorder, higher circulating IL-6 levels have been associated with alterations in sleep architecture, suggesting that low-grade inflammation may already be linked to poorer sleep quality in otherwise healthy populations [44].

Beyond systemic inflammation, sleep disturbance may also induce neuroinflammatory changes within the central nervous system. Experimental work in mice has shown that sleep disturbance can increase hippocampal IL-6 levels, activate microglia, and impair hippocampus-dependent learning and memory [40]. These findings support the view that disrupted sleep may directly contribute to neural dysfunction through localized inflammatory processes rather than simply reflecting a secondary consequence of illness. More broadly, peripheral inflammatory signals can influence the brain, while central immune mediators can in turn modify sleep propensity, sleep intensity, and electroencephalographic activity [41,43]. Altogether, these observations suggest that immune activation and neuroinflammation are integral to the biological mechanisms linking disturbed sleep to cognitive dysfunction and psychiatric vulnerability [40,41,42,43,44].

### 4.4. Microbial Metabolites

**Figure 3:**
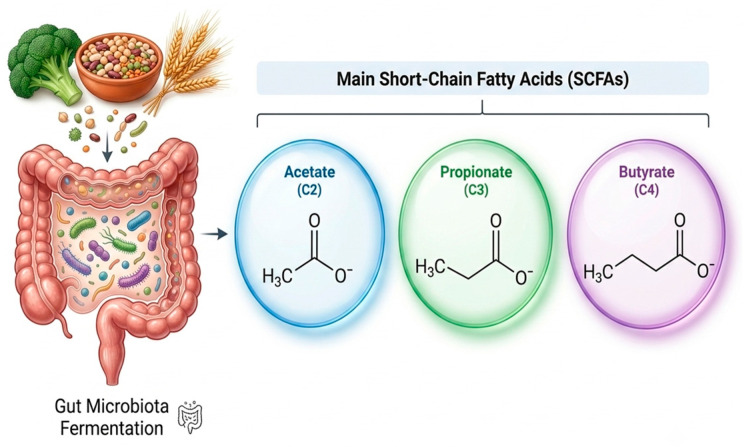
Microbial metabolites involved in gut–brain–sleep communication. Note. The figure summarizes the main microbiota-derived metabolites, including short-chain fatty acids and tryptophan-related pathways, which may contribute to sleep regulation through immune, neuroendocrine, metabolic, and circadian mechanisms. The figure should be interpreted as a mechanistic framework rather than as evidence of direct clinical efficacy.

Microbial metabolites are increasingly recognized as important mediators of gut–brain communication and may represent one of the most direct biochemical pathways through which the intestinal microbiota influences sleep regulation. Among the metabolites most relevant in this context are short-chain fatty acids (SCFAs) and products of tryptophan metabolism, particularly those related to serotonin and melatonin synthesis [45,46,47,48]. Figure 3 provides a schematic representation of the main microbiota-derived metabolic mediators involved in sleep regulation and gut–brain communication.

SCFAs, mainly acetate, propionate, and butyrate, are generated through microbial fermentation of dietary fibers and contribute to intestinal homeostasis, immune regulation, host metabolism, and signaling along the gut–brain axis [45,48]. Recent evidence suggests that altered SCFA profiles may be associated with insomnia and disturbed sleep continuity, although findings are not entirely uniform across populations and study designs [45,48]. In patients with insomnia, lower fecal levels of total SCFAs, acetate, propionate, and butyrate have been reported, together with negative correlations between SCFA concentrations and insomnia severity measures [45]. By contrast, in older adults with insomnia symptoms, higher fecal SCFA concentrations were associated with poorer sleep continuity and a short-sleep insomnia phenotype [48]. These apparently divergent findings should not be interpreted as simple evidence that higher or lower fecal SCFA levels are uniformly beneficial or harmful. Several factors may explain the discrepancy, including differences in age, insomnia phenotype, dietary background, bowel habits, microbiota composition, metabolic status, medication exposure, and comorbid medical conditions. In addition, fecal SCFA concentrations represent the balance between microbial production, intestinal absorption, epithelial utilization, transit time, and fecal excretion. They may therefore not directly reflect luminal production, mucosal exposure, circulating SCFA availability, or signaling at the level of the central nervous system. Higher fecal SCFA levels in some contexts may indicate increased production, but they may also reflect reduced absorption, altered utilization, slower transit, or metabolic dysregulation. Conversely, lower fecal SCFA levels may reflect reduced microbial fermentation, but also more efficient absorption or utilization. Thus, SCFAs remain biologically relevant candidates in the gut–brain–sleep axis, but their interpretation requires integrated assessment of diet, microbiota composition, host metabolism, circulating metabolites, and objective sleep phenotypes.

Tryptophan metabolism provides another important link between the microbiota and sleep. Tryptophan is an essential amino acid and a precursor of serotonin and melatonin, two molecules that play central roles in sleep–wake regulation and circadian timing [46,47]. Serotonin is involved in the regulation of arousal and sleep architecture, whereas melatonin is one of the main hormonal signals coordinating circadian rhythmicity and sleep onset [37,47]. A recent systematic review and meta-analysis found that tryptophan supplementation, particularly at doses of at least 1 g, may improve certain dimensions of sleep quality, most consistently by reducing wake after sleep onset [46]. Microbial and dietary influences on tryptophan metabolism may affect downstream serotonin–melatonin pathways and thereby contribute to sleep continuity, circadian alignment, and vulnerability to insomnia [37,46,47].

## 5. Prebiotics: Definitions and Biological Effects

### 5.1. Types of Prebiotics

Prebiotics are generally defined as non-digestible substrates that are selectively utilized by host microorganisms and confer a health benefit [49]. Although the concept has evolved over time, the compounds most consistently recognized as established prebiotics are carbohydrate-based ingredients that resist digestion in the upper gastrointestinal tract and are fermented by the colonic microbiota [49]. Among these, fructooligosaccharides (FOS), galactooligosaccharides (GOS), inulin, and resistant starch are the most relevant for nutritional and clinical applications [49,50,51,52]. Table 1 summarizes their main sources, microbiota-related effects, and potential relevance to gut–brain signaling.

FOS are short fructose chains linked mainly by β(2→1) bonds, often terminating in a glucose unit, and occur naturally in foods such as onion, garlic, asparagus, banana, chicory, and artichoke [49,52]. Because they are not hydrolyzed by human digestive enzymes, they reach the colon intact, where they are fermented by the intestinal microbiota. FOS are among the most extensively studied prebiotics and are mainly associated with stimulation of beneficial genera such as Bifidobacterium and Lactobacillus [49,52].

GOS are produced mainly from lactose and consist of galactose-containing oligosaccharides with different glycosidic linkages and degrees of polymerization [49,50]. They are well established as prebiotic ingredients and have been widely investigated for their physicochemical properties, physiological effects, and applications in functional foods, especially infant formulas [50]. Similar to FOS, GOS are considered effective substrates for beneficial gut bacteria, particularly bifidobacteria [49,50].

Inulin is a longer-chain fructan, structurally related to FOS but with a higher degree of polymerization, and is commonly derived from plant sources such as chicory root [49,52]. Resistant starch, by contrast, includes starch fractions that escape digestion in the small intestine and are fermented in the large intestine [51]. Although resistant starch has historically been discussed as a dietary fiber rather than a classical prebiotic, growing evidence supports its classification as a candidate or functional prebiotic because of its capacity to promote beneficial microbial growth and short-chain fatty acid production, particularly butyrate [49,51]. These compounds represent the principal prebiotic classes currently used in research and clinical nutrition, differing in structure, fermentability, and selectivity, but sharing the ability to modulate the intestinal ecosystem in potentially beneficial ways [49,50,51,52]. Importantly, prebiotics should not be considered a homogeneous intervention class. FOS, GOS, inulin, resistant starch, and other fermentable substrates differ in molecular structure, degree of polymerization, fermentation rate, site of fermentation, microbial selectivity, and the relative production of acetate, propionate, and butyrate [49,52,53,54,55]. For example, shorter-chain oligosaccharides may be fermented more rapidly, whereas longer-chain fructans or resistant starch may exert more distal colonic effects and promote different cross-feeding interactions [51,53,55]. These differences may influence not only gastrointestinal tolerability, but also downstream immune, metabolic, neuroendocrine, and gut–brain signaling pathways [54,56,57,58,59,60]. Therefore, findings obtained with one prebiotic compound, dose, or formulation cannot be automatically generalized to all prebiotics [49,55]. This is particularly relevant for sleep and psychiatric outcomes, where biological effects may vary according to baseline microbiota composition, habitual diet, host metabolic and gastrointestinal characteristics, concomitant treatments, and the presence or absence of clinically significant sleep disturbance [33,45,48,59,60,61,62].

### 5.2. Mechanisms of Action

The biological effects of prebiotics are mainly mediated through their capacity to reshape the intestinal ecosystem and its metabolic output. Although the precise effects depend on the specific compound, dose, and host context, three mechanisms are consistently emphasized: modulation of gut microbiota composition, production of SCFAs, and support of gut barrier integrity [53,54,55,56,57,58]. This compound-specificity is central to the interpretation of literature. Different prebiotics cannot be assumed to generate equivalent microbial, metabolic, or clinical effects, even when they share the broad property of being fermentable substrates. Consequently, negative findings with one prebiotic do not exclude potential effects of another, while positive findings with a specific formulation should not be generalized to the entire prebiotic category.

By resisting digestion in the upper gastrointestinal tract, prebiotics reach the colon intact, where they are selectively utilized by resident microorganisms [53,57]. This process generally favors the expansion or activity of bacterial groups considered beneficial, particularly Bifidobacterium and Lactobacillus, while also influencing broader community structure and metabolic interactions within the gut microbiota [53,56,57,58]. Rather than acting as simple “fertilizers” for a single bacterial genus, prebiotics appear to promote a functional reorganization of the microbiota, including cross-feeding interactions among microbial populations [53,57,58]. Since altered gut microbial composition has been associated with sleep disturbance, inflammation, and stress-related dysregulation, prebiotic-induced microbial shifts may have downstream effects on neurobiological pathways linked to mental health [33,45,48].

One of the most important consequences of prebiotic fermentation is the generation of SCFAs [53,54,55,56]. These metabolites influence intestinal physiology, immune responses, and host metabolism, and are considered central mediators of gut–brain communication [54,55,56]. Their production depends not only on the presence of prebiotic substrates but also on their physicochemical characteristics and fermentability, which affect the rate and site of fermentation as well as the relative abundance of SCFAs generated [55]. This mechanism is particularly important in the present context because SCFAs have already been linked to sleep continuity and insomnia-related phenotypes, suggesting that prebiotics may influence sleep and psychiatric vulnerability indirectly through microbial metabolite production [45,48]. Accordingly, SCFA generation may represent one of the main pathways through which prebiotic supplementation exerts effects beyond the gut.

Prebiotics may also contribute to the preservation of intestinal barrier function. Current evidence suggests that they can influence epithelial tight junctions both indirectly, through modulation of the microbiota and its metabolites, and possibly also through more direct effects on epithelial signaling [53,54]. Since dysbiosis and increased intestinal permeability are associated with immune activation and systemic inflammation, improvement of gut barrier integrity has been proposed as another mechanism by which prebiotics may exert health benefits [53,54]. This is particularly relevant for psychiatric models in which low-grade inflammation, altered gut permeability, and stress-related neuroimmune activation are increasingly recognized as interacting pathways. Thus, by supporting a more favorable microbial composition, enhancing SCFA production, and stabilizing the gut barrier, prebiotics may influence biological systems that are also implicated in sleep disturbance and psychiatric symptom expression [40,41,42,43,44,45,53,54,55,56,57,58].

### 5.3. Safety and Tolerability

Prebiotics are generally regarded as safe when consumed within the range commonly used in foods or supplements, and their tolerability profile is considered favorable compared with many pharmacological interventions [59,60]. Because they are not digested in the small intestine and are subsequently fermented in the colon, their adverse effects are mainly gastrointestinal and are typically related to osmotic activity and gas production rather than systemic toxicity [59,60].

The most frequently reported side effects are gaseousness, bloating, borborygmi, and abdominal discomfort, while diarrhea tends to occur only at higher doses [59,60]. Tolerance depends not only on the amount consumed but also on the chemical nature of the prebiotic, the rate of fermentation, and individual sensitivity [60]. In general, lower or moderate doses are well tolerated by most healthy individuals, whereas larger intakes may increase the likelihood of unpleasant gastrointestinal symptoms, particularly in subjects with irritable bowel symptoms or heightened visceral sensitivity [59,60]. In addition, some evidence suggests that large daily doses may worsen gastroesophageal reflux in susceptible individuals [60].

Overall, current evidence indicates that prebiotics have a good safety profile, but their clinical use should still consider dose, formulation, and patient characteristics. In the context of psychiatric research, this is particularly important because gastrointestinal tolerability may influence adherence in individuals already experiencing stress-related somatic symptoms or functional gut complaints. Thus, although prebiotics are generally safe, gradual dose escalation and careful consideration of individual tolerance are advisable in both clinical practice and intervention studies [59,60].

## 6. Evidence from Preclinical Studies

### 6.1. Effects on Sleep Architecture

Preclinical evidence specifically addressing the effects of prebiotics on sleep architecture is still limited, but the available data suggest that these compounds may improve sleep under conditions of physiological challenge rather than substantially altering baseline sleep patterns [61,62,63]. In a rat model, dietary supplementation with GOS and polydextrose (PDX) for four weeks did not significantly modify baseline sleep architecture, but it improved sleep responses during repeated sleep disruption and subsequent recovery [63]. Compared with controls, prebiotic-fed rats showed increased N-REM and REM sleep during the period of sleep disruption, as well as greater total sleep time during the recovery phase [63].

These sleep-related changes were accompanied by significant alterations in the fecal microbiome, including increased relative abundance of Parabacteroides distasonis, which positively correlated with recovery sleep parameters [63]. Although the precise mechanisms remain to be clarified, these findings support the hypothesis that prebiotics may enhance resilience of sleep homeostasis through microbiota-dependent pathways. This interpretation is consistent with broader work on the microbiota–gut–brain axis suggesting that microbial modulation can influence sleep-regulatory systems through metabolic, neuroendocrine, and inflammatory signaling [61,62]. Current preclinical findings indicate that prebiotics may not act primarily as direct hypnotic agents, but rather as modulators of sleep stability and recovery in the context of sleep disruption [61,62,63].

### 6.2. Stress Resilience and Behavioral Outcomes

Preclinical studies provide more substantial evidence for the effects of prebiotics on stress-related behavioral phenotypes than on sleep architecture per se. In particular, animal models suggest that prebiotic supplementation may enhance stress resilience and attenuate anxiety-like and depressive-like behaviors [62,64,65]. In mice, chronic administration of FOS and GOS, alone or combined, produced anxiolytic- and antidepressant-like effects across multiple behavioral paradigms, while also reducing stress-induced corticosterone release [64]. Moreover, in animals exposed to chronic psychosocial stress, the FOS + GOS combination attenuated both anxiety-like and depressive-like behaviors, reduced corticosterone elevations, and normalized stress-related alterations in the gut microbiota and pro-inflammatory cytokines [64].

Comparable findings have been reported in rats exposed to acute inescapable stress. Early-life dietary supplementation with GOS, PDX, and/or lactoferrin attenuated stress-induced learned helplessness behaviors later in life, suggesting that prebiotic-related interventions may promote lasting stress resistance [65]. Although these diets did not significantly blunt classic peripheral stress markers such as corticosterone or blood glucose in all cases, they reduced stress-evoked activation within neural circuits strongly implicated in helplessness-like responding, particularly the dorsal raphe nucleus [65]. Taken together, these studies indicate that prebiotics may modulate behavioral responses to stress not only through effects on microbial composition, but also through downstream influences on endocrine, immune, and central neural pathways [62,64,65].

From a psychiatric perspective, these data are especially relevant because they suggest that prebiotics may act on dimensions such as stress vulnerability, emotional reactivity, and depression-like behavior rather than on a single diagnostic phenotype. This supports the view that microbiota-targeted interventions could be particularly valuable in transdiagnostic models of psychiatric risk in which stress sensitivity plays a central pathogenic role [62,64,65].

### 6.3. Neuroinflammation and Neuroplasticity

Beyond behavioral outcomes, preclinical studies suggest that prebiotics may influence neurobiological pathways relevant to psychiatric disorders, particularly neuroinflammation and neuroplasticity [62,64,65,66]. In mice, chronic administration of FOS and GOS not only improved anxiety- and depression-like behaviors but also reduced chronic stress-induced elevations in pro-inflammatory cytokines, supporting the view that prebiotics may buffer the neuroimmune consequences of prolonged stress exposure [64]. These findings are particularly relevant because low-grade inflammation is increasingly recognized as a contributor to emotional dysregulation and stress-related psychopathology.

Prebiotic-related interventions have also been associated with markers of enhanced neuroplasticity. In the rat study by Mika et al., early-life supplementation with GOS, PDX, and lactoferrin altered gene expression in neural circuits central to stress resistance [65]. Notably, the combined diet attenuated stress-induced reductions in 5-HT1A autoreceptor mRNA in the dorsal raphe nucleus and increased basal brain-derived neurotrophic factor (BDNF) mRNA expression in the prefrontal cortex [65]. Because both serotonergic regulation and BDNF signaling are closely linked to adaptive stress responses, synaptic plasticity, and antidepressant mechanisms, these findings suggest that prebiotic supplementation may exert central effects extending beyond microbial or peripheral immune changes.

More broadly, current reviews of the field support the idea that microbiota-targeted interventions may influence neuroplasticity through multiple converging pathways, including microbial metabolite production, immune modulation, neurotransmitter regulation, and trophic signaling [62,66]. Although direct mechanistic evidence remains limited and much of the literature is still preclinical, the available data indicate that prebiotics may modulate neuroinflammation and plasticity-related processes in ways that are potentially relevant to depression, anxiety, and stress-related disorders [62,64,65,66]. This makes them particularly interesting within psychiatric research, where inflammation-related and plasticity-related models increasingly overlap.

Although these preclinical findings provide important mechanistic support, their translational value should be interpreted with caution. Experimental models allow controlled manipulation of diet, stress exposure, microbiota composition, and sleep disruption, but they do not fully capture the heterogeneity of human psychiatric disorders, chronic medication use, lifestyle factors, comorbid medical conditions, or subjective dimensions of sleep disturbance. Thus, preclinical evidence should be considered hypothesis-generating rather than sufficient to infer clinical efficacy.

## 7. Evidence from Human Studies

To improve transparency and facilitate appraisal of the available human evidence, primary clinical studies evaluating prebiotic interventions are presented separately from broader microbiota-targeted evidence. Table 2 summarizes prebiotic clinical trials and reports study design, sample size, comparator, dose, treatment duration, outcomes, main findings, and limitations. Table 3 presents broader evidence from narrative reviews, systematic reviews, meta-analyses, and interventions involving probiotics, synbiotics, paraprobiotics, or mixed microbiota-targeted approaches.

Table 2 and Table 3 highlight the limited number and heterogeneity of primary prebiotic trials, while broader microbiota-targeted reviews provide useful context but cannot be considered direct evidence of prebiotic-specific clinical efficacy.

### 7.1. Effects on Sleep Quality

Human evidence on the effects of prebiotics on sleep quality is still limited, and the available literature suggests that potential benefits are more consistently detected through subjective sleep measures than through objective assessments [67,71,72].

Current human studies on microbiota-targeted interventions suggest some improvement in perceived sleep quality, particularly when evaluated using self-report instruments such as the Pittsburgh Sleep Quality Index (PSQI) [71,72]. Reviews of the field indicate that positive findings are reported more often for subjective outcomes than for objective sleep parameters, although results remain heterogeneous across populations, interventions, and sleep questionnaires [71,72]. A systematic review and meta-analysis on probiotics and paraprobiotics found a modest improvement in PSQI scores, suggesting that perceived sleep quality may be responsive to microbiota-modulating interventions even when other sleep outcomes remain unchanged [72]. Although this evidence is not specific to prebiotics alone, it is relevant because it places prebiotic interventions within a broader microbiota-based clinical framework in which subjective sleep improvements are more readily observed [71,72].

Objective evidence specific to prebiotics is more limited, but one recent randomized, double-blind, placebo-controlled trial evaluated the effects of prebiotic yeast mannan in healthy adults over four weeks and reported significant changes in sleep EEG parameters compared with placebo [67]. In that study, yeast mannan intake was associated with increased non-REM stage 3 (N3) duration, longer total time in bed, and shorter N3 latency, suggesting a possible beneficial effect on deep sleep and sleep architecture [68]. The same trial also identified associations between sleep changes and fecal metabolites, with changes in propionate and GABA emerging as significant explanatory factors for changes in total time in bed and N3 latency, respectively [67]. These findings support the hypothesis that prebiotics may influence objective sleep parameters through metabolite-mediated gut–brain mechanisms [67].

At the same time, the current evidence base remains preliminary [71,72]. Narrative and systematic reviews indicate that objective measures such as actigraphy, polysomnography, or EEG have been used less frequently than self-report questionnaires in human microbiota–sleep studies, and that positive findings have not yet been sufficiently consistent to support firm conclusions [71,72]. In line with this, the available meta-analytic evidence did not show significant overall effects of microbiota-targeted interventions on objective sleep efficiency or latency measures [72]. Overall, the human literature suggests that prebiotics may have beneficial effects on sleep quality, but at present the evidence is stronger for subjective sleep perception than for objective sleep architecture, and larger trials with standardized outcome measures are needed [71,72].

### 7.2. Effects on Stress and Emotional Regulation

Human evidence suggests that prebiotics may influence stress-related neuroendocrine responses and emotional processing, although the number of available studies remains limited [62,68,69,71]. The most direct evidence comes from a randomized, double-blind, placebo-controlled trial in healthy volunteers receiving either FOS, Bimuno^®^-galactooligosaccharides (B-GOS), or placebo for three weeks [68]. In this study, B-GOS supplementation significantly reduced the salivary cortisol awakening response compared with placebo, suggesting a dampening of HPA-axis activity [68]. In addition, participants receiving B-GOS showed decreased attentional vigilance toward negative relative to positive information in an emotional dot-probe task, a pattern interpreted as an early anxiolytic-like cognitive profile [68]. By contrast, FOS did not produce comparable effects, indicating that these responses may depend on the specific prebiotic used rather than reflecting a class effect [68].

These findings are clinically relevant because they suggest that microbiota-targeted interventions may affect not only subjective stress perception, but also cognitive-emotional processes linked to vulnerability for anxiety and affective disorders [62,68,69]. Reviews of the field support this interpretation, indicating that prebiotics may influence emotional regulation through converging pathways involving cortisol modulation, immune signaling, microbial metabolite production, and downstream effects on central neurotransmission [62,69]. More broadly, the emerging literature on biotics and sleep-related outcomes also suggests that reductions in stress burden may accompany improvements in sleep or related psychophysiological measures, further supporting the interconnected nature of these domains within the gut–brain axis framework [71].

It is necessary to recognize that current evidence remains preliminary [62,68,69,71]. Human trials are still few, sample sizes are modest, and the observed effects are more robust for biological stress markers and experimental emotional processing tasks than for broad clinical measures of anxiety or affective symptoms [68,69]. The available evidence suggests that specific prebiotics, particularly B-GOS, may exert beneficial effects on stress regulation and emotional processing in humans, but further trials are needed to determine the reproducibility, magnitude, and clinical relevance of these effects [62,68,69,71].

### 7.3. Effects on Psychiatric Symptoms

Human evidence on the effects of prebiotics on psychiatric symptoms remains limited and, at present, less convincing than the preclinical literature [62,69,70,73,74]. Available data suggest that microbiota-targeted interventions may hold some promise in mood-related disorders, but current findings do not support a clear and consistent benefit of prebiotics alone on clinically relevant depressive or anxiety symptoms [70,73,74].

A key randomized, double-blind, placebo-controlled trial in patients with major depressive disorder compared probiotic, prebiotic, and placebo supplementation over eight weeks [70]. In that study, depressive symptoms measured with the Beck Depression Inventory (BDI) decreased significantly in the probiotic group compared with placebo, whereas the prebiotic intervention did not significantly improve depression scores [70]. Inflammatory cytokines also remained largely unchanged across groups, although urinary cortisol levels decreased in both the probiotic and prebiotic groups relative to baseline, suggesting a possible biological effect without a parallel improvement in depressive symptom severity in the prebiotic arm [70]. These findings indicate that modulation of stress-related physiology does not necessarily translate into short-term clinical improvement in psychiatric symptoms.

This interpretation is supported by meta-analytic evidence. A systematic review and meta-analysis of controlled clinical trials found that prebiotics did not differ from placebo for either depression or anxiety outcomes, whereas probiotics showed small but significant beneficial effects, particularly in clinical or medical samples [71]. More recent systematic reviews focused on psychobiotic interventions in formally diagnosed psychiatric populations similarly conclude that, outside of some encouraging findings in major depression, the current evidence is insufficient to clarify a therapeutic role for prebiotics in mental disorders [70]. Reviews of diet–microbiota–mood interactions also note that although prebiotics may affect emotional and stress-related processes, robust clinical effects on psychiatric symptom scales have not yet been consistently demonstrated [62,70].

The available human literature suggests that prebiotics may influence biological pathways relevant to mental health, but evidence for a direct effect on psychiatric symptoms remains weak and inconsistent [62,69,70,73,74]. At present, prebiotics appear more promising as adjunctive or mechanistically informative interventions than as stand-alone treatments for depression or anxiety. Larger, well-characterized clinical trials in psychiatric populations are needed before firm conclusions can be drawn [70,73,74]. In contrast to the relatively coherent preclinical literature, human evidence remains exploratory and methodologically heterogeneous. Existing studies differ substantially in population characteristics, baseline sleep or psychiatric status, prebiotic type, dose, treatment duration, background diet, concomitant treatments, and outcome measures. In addition, many studies rely primarily on self-reported sleep quality or psychological distress, whereas objective assessments of sleep architecture, circadian phase, microbiome composition, microbial metabolites, inflammation, or HPA-axis function are less consistently included. These limitations reduce the strength of causal inference and make it difficult to identify which patients, prebiotic compounds, and biological pathways are most likely to be clinically relevant.

### 7.4. Limitations of Current Evidence

As already outlined, several limitations constrain the interpretation of current human evidence on prebiotics and related microbiota-targeted interventions in sleep and psychiatric outcomes [67,68,69,70,71,72,73,74].

Many studies have been conducted in relatively small samples, which limits statistical power and increases the risk of both false-negative and unstable positive findings [67,68]. This is particularly relevant in an emerging field where effects may be modest and sensitive to individual differences in baseline diet, microbiota composition, stress exposure, and symptom burden.

The evidence base is highly heterogeneous [71,72,73,74]. Considerable variation exists in the type of intervention studied, including prebiotics, probiotics, synbiotics, paraprobiotics, and mixed nutritional formulations, as well as in the specific compounds administered, dosages, treatment durations, and characteristics of the enrolled populations [67,69,72,74]. Even among prebiotic studies, the compounds used differ substantially, such as B-GOS, FOS, yeast mannan, or galactooligosaccharide-containing products, making direct comparison difficult and limiting the possibility of drawing conclusions about class-wide effects [67,68,70].

Much of the current literature relies predominantly on subjective outcome measures [67,71,72]. In sleep research, improvements are often more apparent on self-report tools such as the PSQI than on objective parameters derived from EEG, actigraphy, or polysomnography [67,71,72]. A similar issue applies to psychiatric outcomes, where self-rated symptom scales frequently predominate and may be more vulnerable to expectancy effects or nonspecific improvements than clinician-rated or biologically anchored endpoints [72,73,74]. Although subjective outcomes remain clinically meaningful, the imbalance between subjective and objective assessments complicates interpretation of the true magnitude and specificity of treatment effects [67,68,69].

Finally, there is still a limited number of trials conducted in well-characterized psychiatric populations, and even fewer specifically examining prebiotics as stand-alone interventions [70,73,74]. As a result, much of the current discussion relies either on broader microbiota-based literature or on small adjunctive studies, making it difficult to determine whether prebiotics have clinically meaningful effects on psychiatric symptoms in their own right [70,73,74]. Summarizing, the present evidence should be considered preliminary, and future studies will need larger samples, better standardization of interventions, and more consistent use of objective and clinically validated outcome measures to clarify the role of prebiotics in sleep and mental health [67,68,69,70,71,72,73,74].

### 7.5. Mechanistic–Clinical Gap in Human Studies

A major limitation of the current human literature is the incomplete connection between the proposed gut–brain–sleep mechanisms and clinically observed outcomes [67,68,69,70,71,72,73,74]. As discussed above, mechanistic evidence implicates HPA-axis activity, cortisol and melatonin rhythmicity, immune-inflammatory signaling, SCFAs, tryptophan-related metabolism, gut barrier integrity, and autonomic pathways in sleep regulation and psychiatric vulnerability [34,35,36,37,38,39,40,41,42,43,44,45,46,47,48,53,54,61,62]. Most human studies assessing microbiota-targeted or prebiotic interventions rely primarily on clinical or questionnaire-based outcomes, such as perceived sleep quality, PSQI scores, emotional processing measures, stress ratings, depressive symptoms, or anxiety-related scales [67,69,70,71,72,73,74]. Only a minority of studies simultaneously assess objective sleep parameters and biological mediators that would allow mechanistic inference [67,68,69,70,71,72,73,74]. The yeast mannan trial by Tanihiro et al. is particularly relevant in this regard because it combined electroencephalographic sleep assessment with fecal metabolite analyses and suggested that changes in propionate and GABA may partly explain selected sleep-related effects [68]. By contrast, most remaining human studies do not integrate objective sleep architecture with microbiome composition, microbial metabolites, inflammatory markers, circadian hormones, or HPA-axis indices [67,69,70,71,72,73,74]. Therefore, although the gut–brain–sleep framework is biologically plausible, the causal pathways linking prebiotic-induced microbiota changes to sleep improvement and psychiatric outcomes in humans remain insufficiently demonstrated [61,62,67,68,69,70,71,72,73,74]. This mechanistic–clinical gap represents one of the most important limitations of the field and should be directly addressed in future trials through integrated designs combining polysomnography or actigraphy, microbiome and metabolomic profiling, neuroendocrine and inflammatory markers, and validated psychiatric endpoints.

## 8. Sleep as a Transdiagnostic Therapeutic Target

**Figure 4:**
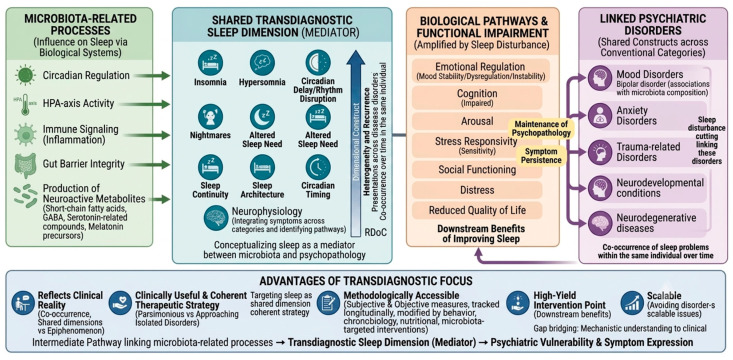
Sleep disturbance as a transdiagnostic dimension across psychiatric disorders. Note. Sleep disturbances, including insomnia, hypersomnia, circadian rhythm disruption, nightmares, and altered sleep need, are common across multiple psychiatric disorders. The figure highlights sleep as a shared dimensional construct linking mood disorders, anxiety disorders, trauma-related disorders, neurodevelopmental conditions, and neurodegenerative diseases, supporting a transdiagnostic and mechanism-based conceptualization of sleep dysfunction in psychiatry.

A dimensional view of psychopathology supports the idea that sleep disturbance should not be considered merely an accessory symptom of individual diagnoses, but rather a process that cuts across traditional diagnostic boundaries [6,75,76]. Sleep problems occur in multiple forms across psychiatric disorders, including insomnia, hypersomnia, circadian delay, nightmares, and reduced sleep need, and they often co-occur within the same individual over time [6,75,76]. This complexity is difficult to accommodate within a strictly categorical model, whereas a transdiagnostic perspective is better suited to capture both the heterogeneity of sleep presentations and their recurrence across disorders [75,76]. Sleep disturbance as a shared transdiagnostic dimension across psychiatric disorders is illustrated in Figure 4.

From this standpoint, sleep may be conceptualized as a mechanistically relevant domain rather than a disorder-specific epiphenomenon [6,75]. Harvey argued that sleep disturbance is present across a wide range of psychiatric conditions and may actively contribute to mood dysregulation, impaired cognition, distress, and reduced quality of life, thereby participating in the maintenance of psychopathology rather than merely reflecting it [75]. More recent work has extended this perspective by emphasizing that sleep and circadian dysfunction may represent a transdiagnostic mechanism linking comorbidity, symptom persistence, and functional impairment across mental disorders [76]. At a broader mechanistic level, sleep neurophysiology has also been proposed as a transdiagnostic framework capable of integrating psychiatric symptoms across conventional diagnostic categories and of identifying therapeutically relevant pathways that cut across disorders [6].

This view is also consistent with dimensional psychiatric models such as the Research Domain Criteria (RDoC) framework, which prioritize domains of functioning and shared mechanisms over rigid diagnostic boundaries [6,76]. If sleep disturbance represents a cross-cutting dimension relevant to emotional regulation, cognition, arousal, and social functioning, then targeting sleep may offer a more coherent therapeutic strategy than approaching each psychiatric disorder in isolation [6,75,76]. This can be considered particularly relevant in clinical reality, where pure diagnostic presentations are relatively uncommon and sleep complaints frequently span multiple symptom dimensions at once [75,76]. Accordingly, evaluating sleep as a transdiagnostic therapeutic target may help bridge mechanistic understanding and clinical intervention in a way that is both conceptually coherent and clinically useful [6,75,76].

Sleep may represent a key intermediate pathway through which microbiota-related processes influence psychiatric vulnerability and symptom expression [6,61,62,67,77]. This hypothesis is supported by converging evidence showing that the gut microbiota can affect sleep through several biological systems already implicated in psychopathology, including circadian regulation, HPA-axis activity, immune signaling, gut barrier integrity, and the production of neuroactive metabolites such as short-chain fatty acids, GABA, serotonin-related compounds, and melatonin precursors [61,62,67,77]. In parallel, sleep disturbance itself is known to amplify emotional dysregulation, cognitive impairment, inflammation, and stress sensitivity, thereby increasing vulnerability across diagnostic categories [6]. From this perspective, sleep is not simply another outcome influenced by the microbiota, but a plausible mechanistic mediator linking gut dysbiosis to broader psychopathological processes [6,61,62,77].

This mediating role helps integrate the evidence reviewed in previous sections into a coherent transdiagnostic model [6,62,67,77]. Microbiota-related changes may alter sleep continuity, sleep architecture, and circadian timing; these sleep disturbances may then contribute downstream to mood instability, impaired stress regulation, and psychiatric symptom persistence [6,77]. Reviews focusing on psychiatric populations suggest that the available human literature is still limited but already points toward meaningful associations between gut microbial composition and sleep quality in disorders such as bipolar disorder, while broader mechanistic reviews support bidirectional interactions between sleep and the microbiota–gut–brain axis [61,67,77]. Therefore, conceptualizing sleep as a mediator between microbiota and psychopathology may offer a clinically useful framework for understanding why microbiota-targeted interventions could influence mental health even when direct effects on psychiatric symptom scales appear modest or inconsistent [6,62,67,77].

A transdiagnostic focus on sleep offers several advantages over disorder-specific approaches [6,75,76]. First, it better reflects clinical reality, where sleep disturbances rarely occur in isolation and often span multiple diagnoses, symptom clusters, and stages of illness [75,76]. Insomnia, hypersomnia, delayed sleep phase, fragmented sleep, nightmares, and reduced sleep need may all appear across different psychiatric disorders and can even coexist within the same individual over time [75,76]. Targeting sleep as a shared dimension may therefore provide a more parsimonious and clinically relevant strategy than attempting to build separate sleep models for each disorder [6,75]. This is also consistent with the already cited dimensional psychiatric approaches (such as RDoC), which prioritize cross-cutting domains of functioning and mechanistic processes over rigid diagnostic boundaries [6,76].

Second, sleep is especially attractive as a therapeutic target because it is both clinically meaningful and methodologically accessible [6,76]. It can be assessed with subjective and objective measures, tracked longitudinally, and modified through behavioral, chronobiological, nutritional, and potentially microbiota-targeted interventions [6,71,76]. Moreover, improvements in sleep may have downstream benefits on emotional regulation, cognition, stress responsivity, and overall functioning, making sleep a potentially high-yield intervention point across disorders [6,75,76]. In contrast, disorder-specific approaches risk overlooking shared mechanisms and may be less scalable in real-world clinical settings, especially given the high rates of comorbidity and the still fragmented evidence base for psychobiotic interventions across individual psychiatric diagnoses [73,75,76]. For these reasons, a sleep-centered transdiagnostic model may offer a more integrative and therapeutically useful framework for future psychiatric research and intervention development [6,75,76].

## 9. Clinical Implications

### 9.1. Evidence Appraisal: From Mechanistic Plausibility to Clinical Translation

A critical appraisal of the available evidence requires a clear distinction between mechanistic plausibility, preclinical findings, and clinical evidence. Mechanistic studies support the biological plausibility of a gut–brain–sleep axis by showing that microbial metabolites, immune-inflammatory signaling, gut barrier integrity, autonomic pathways, and HPA-axis activity may influence sleep regulation and stress-related neurobiology [34,35,36,37,38,39,40,41,42,43,44,45,46,47,48,53,54,55,56,57,58,61,62]. These mechanisms should not be interpreted as direct evidence that prebiotic supplementation improves clinically meaningful psychiatric outcomes in humans [61,62,67,68,69,70,71,72,73,74]. Similarly, preclinical studies provide relatively consistent evidence that selected prebiotics may influence sleep recovery, stress resilience, neuroinflammation, and anxiety- or depression-like behaviors under experimental conditions [61,62,63,64,65,66]. Nevertheless, animal models cannot fully reproduce the complexity of human psychiatric disorders, sleep phenotypes, dietary habits, medication exposure, comorbidities, or interindividual microbiota variability [61,62,66]. Human studies remain fewer, generally small, heterogeneous in terms of prebiotic formulation, dose and duration, and often rely on subjective sleep questionnaires rather than objective sleep measures such as polysomnography or actigraphy [67,68,69,70,71,72,73,74]. Moreover, many trials do not simultaneously assess microbiome composition, microbial metabolites, inflammatory markers, circadian hormones, or HPA-axis indices, limiting the possibility of linking clinical changes to the proposed biological mechanisms [67,68,69,70,71,72,73,74]. Therefore, the current evidence supports prebiotics as biologically plausible and generally well-tolerated adjunctive interventions [59,60,61,62], but it does not yet support their use as stand-alone treatments for insomnia or psychiatric disorders [67,68,69,70,71,72,73,74]. Clinical claims should remain cautious until adequately powered randomized controlled trials demonstrate consistent benefits on objective sleep architecture, validated psychiatric outcomes, and integrated microbiome–metabolite endpoints.

Several factors may contribute to the inconsistency between preclinical findings and human trials. In animal studies, genetic background, diet, housing conditions, stress exposure, sleep disruption, and microbiota composition can be tightly controlled, whereas human studies are affected by substantial interindividual variability [61,62,63,64,65,66]. Baseline microbiota composition may determine whether a given prebiotic can be efficiently fermented and whether specific microbial metabolites are produced [53,54,55,56,57,58]. Similarly, differences in dose, formulation, intervention duration, and habitual fiber intake may modify microbial fermentation and metabolite production [49,50,51,52,53,54,55,56,57,58]. Medication use, psychiatric comorbidity, gastrointestinal symptoms, and adherence may further influence tolerability and clinical response [59,60,67,68,69,70,71,72,73,74]. Population heterogeneity is also critical: findings obtained in healthy volunteers or mildly stressed individuals may not translate to patients with major depression, bipolar disorder, post-traumatic stress disorder, chronic insomnia, or neurodevelopmental conditions [67,68,69,70,71,72,73,74]. These factors may explain why preclinical studies often show clearer effects on stress resilience, neuroinflammation, and sleep recovery, whereas human trials have produced more variable and frequently modest results.

### 9.2. Integration with Current Treatments

In routine psychiatric care, sleep disturbances are often managed pharmacologically with hypnotics, sedating antidepressants, melatonin-related agents, wake-promoting drugs, or other compounds selected according to the predominant sleep complaint and the broader clinical context [78,79]. However, both classical and more recent reviews of sleep pharmacology make clear that these treatments are mainly symptom-oriented and require careful risk–benefit evaluation because of adverse effects, residual daytime sedation, dependence liability, cognitive side effects, and drug–drug interactions, particularly in patients with psychiatric comorbidity [78,79]. From the perspective developed in this review, sleep-focused and microbiota-informed strategies should therefore be understood not as substitutes for standard pharmacotherapy, but as potentially useful adjuncts. This may be especially relevant when residual insomnia, circadian disruption, or non-restorative sleep persists despite adequate psychiatric treatment, or when improving sleep could enhance overall emotional regulation and daytime functioning across diagnoses [6,75,76]. In this sense, integrating sleep as a therapeutic target may strengthen conventional pharmacological care by addressing a transdiagnostic process that often remains only partially treated [6,78,79].

Among current nonpharmacological treatments, Cognitive Behavioral Therapy for Insomnia (CBT-I) is viewed as the most established intervention for insomnia and is particularly relevant for psychiatric populations [75,76]. Sleep disturbance is treated not merely as secondary to mental illness, but as a clinically meaningful therapeutic target in its own right [75]. More recent work extends this view by showing that sleep and circadian interventions can be framed transdiagnostically across a range of psychiatric disorders and heterogeneous sleep presentations [76]. Within this framework, microbiota-targeted strategies such as prebiotic supplementation are unlikely to replace CBT-I but may plausibly complement it. A combined approach may be particularly useful in patients whose insomnia coexists with stress dysregulation, circadian instability, inflammatory vulnerability, or diet-related risk factors [6,62,76,77]. Accordingly, the most realistic clinical implication at present is not a replacement model, but an integrative one in which established behavioral sleep therapies remain central and microbiota-focused interventions are explored as potential adjuncts to established treatment [62,76,77].

**Figure 5:**
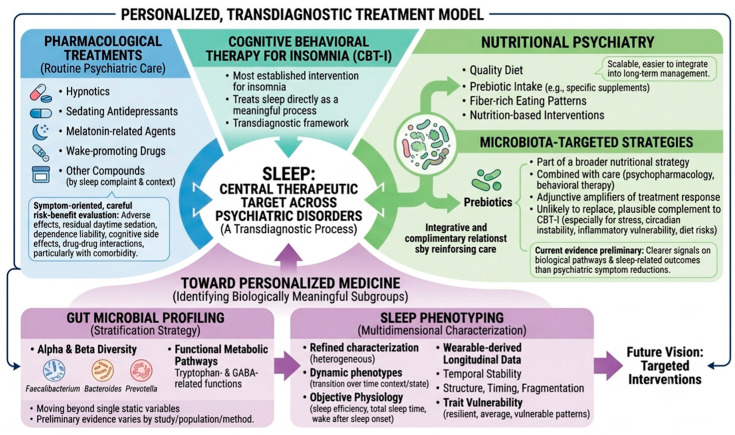
Clinical framework integrating sleep interventions and microbiota-targeted strategies in psychiatry. Note. The figure presents a clinical framework integrating pharmacological treatments, cognitive behavioral therapy for insomnia, nutritional psychiatry, and microbiota-targeted interventions. Sleep is positioned as a central therapeutic target across psychiatric disorders, with prebiotics and related strategies acting as adjunctive modulators within a personalized, transdiagnostic treatment model.

Nutritional psychiatry provides a particularly suitable frame of reference for integrating sleep, microbiota modulation, and mental health treatment [62,71,77]. Dietary quality, prebiotic intake, fiber-rich eating patterns, and other nutrition-based interventions may influence gut microbial composition, inflammatory signaling, metabolite production, and circadian regulation, all of which are relevant to both sleep and psychopathology [62,71,77]. In this context, prebiotics are best conceptualized not as isolated supplements, but as part of a broader nutritional strategy that may be combined with routine psychiatric care, psychopharmacology, and behavioral sleep treatment [71,77]. This approach may be clinically attractive because nutritional interventions are generally more scalable and easier to integrate into long-term management than highly specialized microbiome procedures. At the same time, the current evidence base remains preliminary, and available studies suggest that the clearest signals concern biological pathways and sleep-related outcomes rather than robust reductions in psychiatric symptoms per se [62,71,77]. Even so, the convergence between nutritional psychiatry and transdiagnostic sleep models supports the idea that sleep may be one of the most practical clinical entry points through which diet- and microbiota-focused strategies can be incorporated into psychiatric treatment [6,71,77]. A potential clinical implementation framework integrating sleep-focused and microbiota-targeted strategies is shown in Figure 5.

### 9.3. Toward Personalized Medicine

A personalized medicine approach in this field will likely depend, at least in part, on the ability to identify biologically meaningful subgroups based on both gut microbial features and longitudinal sleep-related patterns [77,80,81,82,83]. Recent work suggests that clinically useful stratification may require moving beyond single static variables and instead integrating dynamic sleep phenotypes with microbiota-related signatures [80]. In parallel, accumulating evidence indicates that the relationship between the gut microbiome and sleep is not reducible to a single universal pattern, but may involve differences in microbial composition, functional metabolic pathways, inflammatory signaling, and host-specific factors such as circadian disruption, diet, and comorbid disease [77,81]. Human studies have linked sleep quality to specific taxa and pathways, including Faecalibacterium, Bacteroides, Prevotella, and tryptophan- or GABA-related metabolic functions, supporting the idea that microbiota profiling may eventually help identify biologically meaningful subgroups of patients [81,82,83]. At the same time, the evidence remains preliminary: some studies report associations with alpha diversity, others with beta diversity or specific taxa, and findings vary according to the population studied and the method used to assess sleep [81,82,83]. Therefore, microbiota profiling should currently be viewed less as a ready-to-use clinical tool and more as a promising stratification strategy that may help future interventions become more targeted, especially if integrated with sleep, metabolic, and psychiatric phenotyping [77,80,81,82,83].

Personalized approaches will also require a more refined characterization of sleep itself [76,80,82,84]. Sleep disturbance in psychiatry is highly heterogeneous, and patients may differ not only in symptom severity but also in the structure, timing, fragmentation, and temporal stability of their sleep [76,80]. Recent large-scale work using wearable-derived longitudinal data suggests that individuals transition across different sleep phenotypes over time, and that these transition patterns may carry clinically relevant information beyond static sleep classification alone [80]. This is particularly important because it supports a shift from viewing sleep only as a single score or diagnosis toward understanding it as a dynamic phenotype that may vary within the same individual across contexts, illness states, and treatment phases [80]. Other studies further indicate that objective sleep physiology, including sleep efficiency, total sleep time, and wake after sleep onset, may be associated with microbiome diversity and inflammatory markers, reinforcing the potential value of multidimensional sleep phenotyping [82]. More broadly, earlier work on trait vulnerability to sleep loss also supports the existence of relatively stable inter-individual phenotypes, such as resilient, average, and vulnerable patterns, which may be relevant when considering personalized sleep interventions [84]. Individualized sleep phenotyping could become a key component of precision psychiatry, especially if combined with microbiota profiling and transdiagnostic clinical assessment [76,80,82,84,85]. Beyond microbiota and sleep-related markers, future personalized approaches may incorporate digital phenotyping strategies capable of capturing behavioral and cognitive features through passive or task-based assessments. Machine learning models applied to multimodal digital signals, including handwriting and speech characteristics, have shown preliminary potential as complementary tools for psychiatric assessment. Although further validation is required, these approaches may eventually be integrated with biological, microbiota, and sleep-related measures to improve patient stratification and support precision psychiatry frameworks [86,87,88].

### 9.4. Clinical Applicability: Adjunctive Use and Practical Considerations

From a clinical perspective, prebiotics should currently be considered adjunctive nutritional strategies rather than primary treatments for insomnia or psychiatric disorders [61,62,67,68,69,70,71,72,73,74]. Their potential use may be most appropriate within multimodal care plans that include evidence-based psychiatric treatment, sleep hygiene, chronotherapeutic approaches when indicated, and cognitive behavioral therapy for insomnia. In this framework, prebiotics may be explored as supportive interventions aimed at modulating gut–brain–sleep pathways, particularly in populations characterized by poor diet quality, low fiber intake, stress-related sleep disturbance, or gastrointestinal complaints [49,53,54,55,56,57,58,61,62]. However, clinicians should avoid presenting prebiotics as substitutes for pharmacotherapy, psychotherapy, CBT-I, or disorder-specific interventions [67,68,69,70,71,72,73,74].

Practical implementation should consider compound type, dose, duration, adherence, and tolerability [49,50,51,52,53,54,55,56,57,58,59,60]. Since available studies have used heterogeneous formulations and protocols, no single prebiotic, dose, or treatment duration can currently be recommended for psychiatric or sleep indications [67,68,69,70,71,72,73,74]. In general, gradual introduction and dose escalation may improve tolerability, especially in patients prone to bloating, abdominal discomfort, irritable bowel symptoms, or functional gastrointestinal complaints [59,60]. Treatment duration should also be interpreted cautiously: short trials may be sufficient to detect changes in microbial metabolism or subjective sleep quality, but longer follow-up is needed to determine whether effects are sustained and clinically meaningful [67,68,69,70,71,72,73,74]. Monitoring should include not only sleep quality and psychiatric symptoms, but also gastrointestinal adverse effects, adherence, dietary background, and concomitant treatments.

Prebiotics may also be considered in combination with CBT-I or other conventional psychiatric treatments, but such integration remains largely theoretical and requires empirical validation [61,62,67,68,69,70,71,72,73,74]. A possible adjunctive model would involve baseline assessment of sleep symptoms, diet quality, gastrointestinal tolerance, medication use, and psychiatric severity; selection of a specific prebiotic formulation with gradual titration; monitoring of subjective and, where feasible, objective sleep outcomes; and continuation only when tolerability and clinically meaningful benefit are observed. This pragmatic approach may help avoid premature therapeutic claims while allowing future studies to test whether prebiotics can enhance response to established sleep-focused and psychiatric interventions.

**Figure 6:**
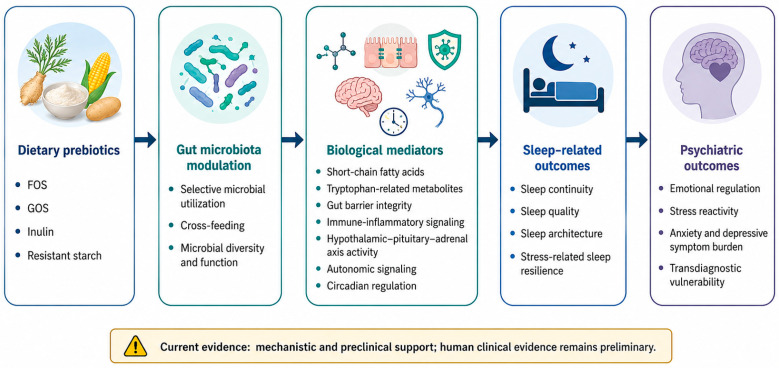
Proposed conceptual framework and hypothesized mechanistic pathway linking prebiotics, sleep regulation, and psychiatric outcomes. Note. The figure summarizes the central hypothesis of the review. Dietary prebiotics may selectively modulate resident gut microbial communities and their metabolic output, including short-chain fatty acids and tryptophan-related metabolites. It is proposed that these changes may influence gut barrier integrity, immune-inflammatory signaling, hypothalamic–pituitary–adrenal axis activity, autonomic pathways, and circadian regulation. Through these mechanisms, it is hypothesized that prebiotics may contribute to improved sleep continuity, sleep quality, and stress-related sleep regulation, which in turn may indirectly influence psychiatric symptom burden. This pathway remains hypothetical and should be interpreted as a framework for future research rather than as an established clinical treatment model.

The proposed clinical pathway linking prebiotics, sleep regulation, and psychiatric outcomes is summarized in Figure 6. This model should be interpreted as a hypothesis-generating framework. It integrates mechanistic and preclinical evidence with preliminary human findings, but it does not imply that prebiotics have established efficacy as stand-alone treatments for insomnia or psychiatric disorders.

## 10. Limitations and Future Research Directions

Several limitations should be considered when interpreting the current evidence on prebiotics, sleep, and psychiatric outcomes. Much of the mechanistic support derives from preclinical studies, which provide valuable biological insight but cannot fully reproduce the complexity of human psychiatric disorders, chronic sleep disturbance, diet, medication exposure, comorbidities, and interindividual microbiota variability [61,62]. In addition, human intervention studies remain relatively few and often include small samples, short intervention periods, and heterogeneous populations, ranging from healthy volunteers to individuals with psychological distress or diagnosed psychiatric disorders [67,68,69,70,71,72,73,74]. Prebiotic interventions differ substantially in compound type, dose, formulation, duration, fermentability, and expected metabolic effects, making it difficult to compare studies or identify an optimal intervention protocol [49,50,51,52,53,54,55,56,57,58]. Microbiome analyses are not standardized across studies, with differences in sequencing methods, taxonomic resolution, bioinformatic pipelines, and metabolomic assessment limiting cross-study comparability. Sleep outcomes are frequently assessed using subjective questionnaires, whereas objective measures such as polysomnography, actigraphy, circadian phase markers, or electroencephalographic sleep architecture are less consistently included. Many human studies do not simultaneously assess microbiome composition, microbial metabolites, inflammatory markers, HPA-axis activity, or circadian hormones, thereby limiting mechanistic interpretation [67,68,69,70,71,72,73,74]. Finally, responder profiles remain poorly characterized, and it is still unclear whether benefits are more likely in individuals with low habitual fiber intake, specific baseline microbiota configurations, gastrointestinal symptoms, stress-related hyperarousal, or clinically significant insomnia. These limitations indicate that the available evidence should be considered preliminary and hypothesis-generating rather than sufficient for definitive clinical recommendations.

Ongoing research should move beyond small, heterogeneous proof-of-concept studies and adopt more rigorous and integrated designs capable of clarifying whether prebiotics exert clinically meaningful effects on sleep and psychiatric outcomes [67,68,69,72,74,85]. In particular, randomized controlled trials should combine validated subjective instruments with objective sleep measures such as actigraphy, polysomnography, or EEG-based assessments, in order to determine whether microbiota-targeted interventions influence not only perceived sleep quality but also sleep architecture, continuity, and circadian timing [67,68,69,85]. This appears especially important given that current human evidence is stronger for subjective than for objective sleep outcomes [67,68,69], and that recent methodological work in insomnia research has further emphasized the need for more high-quality randomized trials and broader use of objective sleep measures when evaluating behavioral and sleep-focused interventions [85]. These studies should compare different formulations, doses, and intervention durations to clarify dose–response relationships and identify the minimum effective exposure required to influence sleep-related and psychiatric outcomes.

Future trials should incorporate microbiome sequencing and, where possible, functional readouts such as metabolite profiling or inflammation-related biomarkers [77,81,82,83]. Existing evidence suggests that associations between sleep and the gut microbiome may depend not only on taxonomic composition, but also on metabolic pathways involving tryptophan, GABA-related processes, short-chain fatty acids, and other signaling systems relevant to the gut–brain axis [77,81,82,83]. Integrating objective sleep measures with microbial and metabolic profiling would therefore allow a more mechanistic understanding of treatment effects and help identify which biological pathways are most relevant to sleep improvement [77,81,82,83].

Dose–response studies are also needed. Current trials vary widely in the type, dose, and duration of prebiotic administration, making it difficult to determine optimal treatment parameters or compare findings across studies [72,74]. Establishing whether clinical effects depend on dose, treatment length, or baseline patient characteristics will be essential before prebiotic interventions can be translated more confidently into psychiatric practice [72,74].

Longitudinal designs should likewise become a priority [80,81,82,83]. Sleep, microbiota composition, and psychiatric symptoms are all dynamic phenomena, and cross-sectional or short-duration studies are unlikely to capture their reciprocal interactions adequately [80,81]. Longitudinal approaches may be particularly informative for identifying sleep phenotypes, microbiota-related trajectories, and time-dependent treatment responses, thereby improving both mechanistic interpretation and clinical stratification [80,82,83].

Finally, future research should also explore synbiotic interventions, combining prebiotics with probiotics, as these may produce broader or more stable effects than either approach alone [72,74]. The potential clinical value of synbiotic combinations should be tested separately from prebiotics alone, because combining substrates with selected bacterial strains may produce different biological and clinical effects.

Trials should evaluate prebiotics as adjunctive interventions combined with established treatments, including CBT-I, pharmacotherapy, psychotherapy, chronotherapeutic approaches, and lifestyle interventions, to determine whether they can improve adherence, sleep continuity, stress regulation, or psychiatric outcomes beyond standard care.

Current evidence in psychiatric populations remains preliminary and does not yet allow firm conclusions, but the relative weakness of prebiotic-only effects on psychiatric symptoms suggests that combined microbiota-targeted strategies may deserve closer investigation [72,74]. The next generation of studies should aim to be multimodal, longitudinal, and biologically informed, so that sleep can be evaluated not only as an outcome, but also as a mechanistic and transdiagnostic target linking the gut microbiome to mental health [76,77,80,81,82,83,85].

## 11. Conclusions

Sleep represents a clinically relevant transdiagnostic dimension in psychiatry and a promising framework for investigating microbiota-targeted interventions. Prebiotics may influence biological pathways involved in sleep regulation, stress responsivity, inflammation, and gut–brain communication, but current human evidence remains limited and heterogeneous. At present, prebiotics should be regarded as potential adjunctive nutritional strategies rather than established treatments for insomnia or psychiatric disorders. Further well-designed clinical trials are required to determine whether specific prebiotic interventions can produce reproducible and clinically meaningful benefits.

## Figures and Tables

**Table 1:** Main prebiotic compounds and their potential relevance to the gut–brain–sleep axis.

Prebiotic Compound	Main Sources/Origin	Main Microbiota-Related Effects	Potential Relevance
Fructooligosaccharides (FOS)	Onion, garlic, asparagus, banana, chicory, artichoke	Stimulate beneficial bacteria, especially *Bifidobacterium* and *Lactobacillus*	Microbiota modulation and gut–brain signaling
Galactooligosaccharides (GOS)	Produced mainly from lactose	Promote bifidobacteria and beneficial microbial activity	Stress regulation and emotional processing
Inulin	Chicory root and other plant sources	Fermented by gut microbiota; supports beneficial microbial activity	SCFA production and gut barrier support
Resistant starch	Starch fractions reaching the colon undigested	Promotes microbial fermentation and butyrate production	Immune regulation and gut–brain communication

Abbreviations: SCFA, short-chain fatty acid.

**Table 2:** Primary human clinical studies evaluating prebiotic interventions in relation to sleep, stress-related, and psychiatric outcomes.

Study	Study Design	Population and Sample Size	Prebiotic Intervention	Comparator/Control	Dose	Duration	Outcomes Assessed	Main Findings	Main Limitations
Tanihiro et al. [67]	Randomized, double-blind, placebo-controlled, parallel-group trial	40 healthy Japanese adults aged 22–64 years with discomfort related to defecation; 20 participants per group	Yeast mannan	Placebo in which yeast mannan was replaced with maltose	1.1 g/day of yeast mannan, providing 0.62 g/day of mannan	4 weeks	Home electroencephalography-based sleep parameters; bowel habits; fecal microbiota and metabolomics	Yeast mannan was associated with increased N3 duration, longer total time in bed, and shorter N3 latency. Exploratory analyses identified changes in fecal propionate and gamma-aminobutyric acid as possible explanatory factors for selected sleep changes	Small, healthy sample selected for defecation discomfort; sleep was not the primary outcome; short intervention; exploratory metabolomic analyses; no psychiatric outcomes
Schmidt et al. [68]	Randomized, double-blind, placebo-controlled, three-arm trial	48 healthy adults enrolled; 45 completed and were analyzed: 15 B-GOS, 15 FOS, and 15 placebo	Bimuno galactooligosaccharides or fructooligosaccharides	Maltodextrin placebo	5.5 g/day of B-GOS or 5.5 g/day of FOS	3 weeks	Salivary cortisol awakening response; emotional-processing tasks; subjective anxiety and stress measures	B-GOS, but not FOS, reduced the cortisol awakening response and altered attentional bias toward emotional information compared with placebo. No significant effects were observed on subjective anxiety or perceived stress	Small healthy sample; short intervention; no sleep outcomes; no psychiatric diagnosis; findings were specific to B-GOS and cannot be generalized to all prebiotics
Johnstone et al. [69]	Randomized, double-blind, placebo-controlled, parallel-group trial	64 healthy females aged 18–25 years	Galactooligosaccharide prebiotic	Maltodextrin/dried glucose syrup placebo	7.5 g/day of powder, providing approximately 5.5 g/day of GOS	28 days	State and trait anxiety; depression; mood; emotion regulation; attentional bias; Pittsburgh Sleep Quality Index; fecal microbiota composition	GOS increased the relative abundance of *Bifidobacterium*. Psychological effects were mainly observed among participants with high baseline trait anxiety, including reduced negative attentional bias and suggestive improvement in trait-anxiety indices. No clear overall improvement was found in mood, depression, state anxiety, or sleep quality	Healthy young female sample; subgroup-dependent and partly trend-level findings; multiple outcomes; no objective sleep assessment; limited generalizability to psychiatric populations
Kazemi et al. [70]	Randomized, double-blind, placebo-controlled, three-arm clinical trial	110 adults with major depressive disorder randomized; 81 completed the study	Galactooligosaccharide prebiotic; a separate probiotic arm was also included	Placebo	5 g/day of GOS	8 weeks	Beck Depression Inventory; kynurenine/tryptophan ratio; tryptophan/branched-chain amino-acid ratio; inflammatory and neuroendocrine markers assessed in related analyses	The prebiotic intervention did not significantly improve depressive symptoms compared with placebo. The more consistent improvement in depressive symptoms was observed in the probiotic arm. Prebiotic-related effects on biological markers were limited and inconsistent	Attrition; relatively small final sample across three groups; concomitant antidepressant treatment; no sleep outcomes; microbiome composition not directly assessed; difficult to isolate clinically meaningful prebiotic-specific effects

Abbreviations: B-GOS, Bimuno galactooligosaccharides; FOS, fructooligosaccharides; GOS, galactooligosaccharides; N3, stage 3 non-rapid eye movement sleep.

**Table 3:** Broader human evidence from reviews and microbiota-targeted interventions relevant to sleep and psychiatric outcomes.

Study	Evidence Type	Evidence Included	Intervention Categories	Outcomes Assessed	Main Findings	Relevance to the Present Review	Main Limitations
Haarhuis et al. [71]	Narrative review	Human and mechanistic evidence relevant to sleep quality	Probiotics, prebiotics, and postbiotics	Subjective and objective sleep outcomes; microbial metabolites and potential sleep-regulatory mechanisms	The review identified biologically plausible pathways through which microbiota-targeted interventions might influence sleep, but emphasized that direct human evidence was limited and heterogeneous	Provides a broad conceptual framework for microbiota–sleep interactions	Narrative design; heterogeneous interventions; limited prebiotic-specific clinical evidence; no pooled estimate
Irwin et al. [72]	Systematic review and meta-analysis	14 studies comprising 20 controlled trials in adults	Probiotics and paraprobiotics	Subjective and objective sleep metrics	Probiotic and paraprobiotic interventions were associated with modest improvements in selected subjective sleep measures, whereas findings for objective sleep outcomes were less consistent	Provides contextual evidence that microbiota-targeted interventions may affect sleep, but does not establish prebiotic efficacy	No prebiotic-specific analysis; substantial heterogeneity in strains, populations, duration, and sleep measures; limited objective sleep data
Ribera et al. [73]	Systematic review of clinical trials	Clinical trials conducted in psychiatric populations	Probiotics, prebiotics, synbiotics, and fermented foods	Psychiatric symptoms and related biological or metabolic outcomes	The review found increasing interest in microbiota-targeted interventions across psychiatric disorders, but the findings were heterogeneous and insufficient to support firm clinical recommendations	Places prebiotic evidence within the wider field of nutritional psychiatry and psychobiotics	Broad diagnostic and intervention heterogeneity; few prebiotic-only trials; variable comparators, formulations, doses, and outcomes
Liu et al. [74]	Systematic review and random-effects meta-analysis	34 controlled clinical trials	Prebiotics and probiotics	Depression and anxiety outcomes	Probiotics showed more consistent effects on depressive symptoms, whereas prebiotics did not significantly differ from placebo for either depression or anxiety	Provides direct comparative context showing that evidence for prebiotic-only psychological effects is weak	Limited number of prebiotic trials; heterogeneous populations and interventions; psychological outcomes were often secondary endpoints; no specific focus on sleep

## Data Availability

No new data were created or analyzed in this study. Data sharing is not applicable to this article.
